# Evaluation of Physical, Mechanical and Antibacterial Properties of Pinto Bean Starch-Polyvinyl Alcohol Biodegradable Films Reinforced with Cinnamon Essential Oil

**DOI:** 10.3390/polym13162778

**Published:** 2021-08-18

**Authors:** Ali Khazaei, Leila Nateghi, Nazanin Zand, Abdulrasoul Oromiehie, Farhad Garavand

**Affiliations:** 1 Department of Food Science and Technology, Faculty of Agriculture, Varamin-Pishva Branch, Islamic Azad University, Varamin 3381774895, Iran; alikhazaei777@yahoo.com (A.K.); n_zand2008@yahoo.com (N.Z.); 2 Department of Polymer Engineering, Faculty of Engineering, Southern Tehran Branch, Islamic Azad University, Tehran 1584743311, Iran; oromia2000@yahoo.com; 3 Department of Food Chemistry and Technology, Teagasc Food Research Centre, Moorepark, Fermoy, P61 C996 Cork, Ireland; farhad.garavand@teagasc.ie

**Keywords:** pinto bean starch, polyvinyl alcohol, packaging film, essential oils, antimicrobial properties

## Abstract

In the present study, various blended films from polyvinyl alcohol (PVA) and pinto bean starch (PBS) were prepared and the selected film was used to fabricate an antimicrobial packaging film. Different essential oils (EOs) were also exposed to minimum inhibitory concentration (MIC) and minimum bactericidal concentration (MBC) tests to find the most efficient EO against a range of microorganisms. From the primary studies, the PVA:PBS (80:20) and cinnamon essential oil (CEO) were chosen. Afterward, the blend composite film reinforced by 1, 2, and 3% CEO and several, physical, mechanical, structural, and antimicrobial attributes were scrutinized. The results showed a significant modification of the barrier and mechanical properties of the selected blended films as a result of CEO addition. Scanning electron micrographs confirmed the incorporation and distribution of CEO within the film matrix. The X-ray diffraction (XRD) patterns and Fourier transform infrared (FTIR) spectra indicated the interaction of CEO and the PVA-PBS composite. The antibacterial of the tested bacteria showed a significant increase by increasing the CEO concentration within the control film. CEO-loaded films were more effective in controlling Gram-positive bacteria compared to Gram-negative bacteria. It can be concluded that PVA-PBS-CEO films are promising candidates to produce biodegradable functional films for food and biomedical applications.

## 1. Introduction

In recent decades, diseases caused by foods contaminated with spoilage and pathogenic bacteria have become a serious concern for consumers. The use of chemical antimicrobials to prevent or delay food spoilage is widely used today. There have been several debates about the carcinogenicity, mutagenicity, and toxicity effects of synthesized chemical preservatives [1]. For this reason, food producers and consumers should be cautious enough in using such preservatives. There are many factors to consider when designing an antimicrobial package; most of these factors depend on the chemical nature of the antimicrobial agent, the type of food, and the target microorganism [2]. Recently, biopolymers derived from biological sources have shown great potential as safe, abundant, ecofriendly, and economic substitutes for the synthetic polymers derived from petroleum sources, and developed as edible packaging materials for food industry applications [3]. Active packaging is defined as a type of packaging that changes the packaging conditions favorably and increases the shelf life of the food. Antimicrobial packaging is also a type of active packaging that, in interaction with the closed space, reduces, inhibits or delays the growth of microorganisms that may be present on food surfaces [4,5].

Recently, various biodegradable packaging films and coatings have been designed based on carbohydrates, proteins and, to some extent, fats (waxes) to fabricate biodegradable film composites for food and biomedical uses [6]. Of these, carbohydrate-based composites have attracted tremendous attention due to their abundance and cost-effectiveness along with the fact that most of them have been obtained from agrofood byproducts [7]. Starch, cellulose, pectin, chitosan, and gums, in addition to their derivatives, are the main carbohydrate-based composites used in the food packaging field so far [8]. Among the abovementioned biopolymers, starches are considered as suitable film-forming biocomposites owing to their high extraction efficiency, availability, biodegradability, biocompatibility, and economical concerns [9]. Starch-based composite films are semi-permeable barriers against various gases (e.g., oxygen and carbon dioxide), moisture, flavors, and lipid components, and have no toxicity, odor, color, taste, etc. Starch is not considered as a real thermoplastic biopolymer, while the addition of water and/or combination with other polymers can construct a thermoplastic network [10]. It has been reported that water addition can induce the mobility of starch chains, then the following heat process at high temperatures (>90 °C) can also disrupt the three-dimensional structure and semi-crystallinity of starch granules [9]. Pinto beans are classified as pulses and considered as one of the potential budget-friendly sources of starch (containing 17–20%). Pinto bean starch (PBS) has exceptional attributes, such as higher elasticity, shear resistance, and gelation temperature, compared to other starch sources, mainly due to its higher amylose content [11]. However, various operational drawbacks, such as low thermo-mechanical strength, limited gas barrier, rigidity, and weak mechanical strength attributes are associated with starch film composites in general [12]. Such limitations can be improved by various strategies including combination with polymers, chemical modifications, and the addition of plasticizers, crosslinking agents, nanofillers, etc. Blending starch with synthetic polymers is one of the straightforward methods to strengthen the structural properties of starch composite films [8,13]. The combination of different starches with polyvinyl alcohol (PVA) has been widely scrutinized by several researchers to fabricate a biobased composite with elevated operational characteristics [14,15,16]. PVA is recognized as a non-toxic, biodegradable, synthetic, and water-soluble polymer derived from polyvinyl acetate [15]. It has excellent film-forming properties such as fabricating composite films possessing high mechanical strength, proper gas barrier attributes, flexibility, and acid–alkaline resistance [17]. PVA also showed high miscibility with hydrophilic nature biopolymers such as starch, chitosan, etc., mainly due to the formation of intermolecular hydrogen bonds [14,17]. Over the last few years, packaging systems fitted out with antimicrobial agents have been designed to extend the shelf life of perishable food products. Essential oils (EOs) and other bioactive substances are considered to be food-grade natural antimicrobial agents capable of extending the food shelf life in a safe manner. Various EOs (e.g., cinnamon, bergamot, oregano, ginger, lemon, etc.) have been employed to improve the functional and antimicrobial characteristics of food packaging composites [18,19,20,21].

Herein, the main purpose of the current research is to find the best PVA:PBS ratio, and the type of EOs (i.e., cinnamon essential oil (CEO), ginger essential oil (GiEO) and garlic essential oil (GaEO)) in order to fabricate active biocomposite films. The synthesized blend films were subjected to various physical (solubility, opacity, and barrier properties), mechanical, structural (scanning electron microscopy (SEM) and X-ray diffraction (XRD)), and antimicrobial (agar diffusion) tests in addition to Fourier transform infrared (FTIR) to monitor the interactions that occurred between film components.

## 2. Materials and Methods

### 2.1. Chemicals and Raw Materials

Pinto beans were purchased from a local market in Tehran (Tehran, Iran). PVA pellets (purity 98%, molecular weight 105,000, and degree of hydrolysis 97%) were supplied from GC Co. (Guangdong, China). Dimethyl sulfoxide (DMSO), tryptone soya agar (TSA), and Muller-Hinton broth (MHB) were supplied by Sigma-Aldrich (London, UK). Microorganisms were also obtained from Persian Type Culture Collection (PTCC) (Tehran, Iran). Other chemicals and reagents were purchased from Merck Co. (Darmstadt, Germany).

### 2.2. PBS Extraction

PBS was separated from pinto beans according to the procedure described by Betancur et al. [22]. Accordingly, impurities were discarded from pinto beans first, then the separated beans were milled by a Moulinex Multi-Grinder (Model K6A8, Paris, France), and the obtained flour was passed through a 20 mesh sieve. Afterward, the obtained flour was mixed with water in a ratio of 1:10 and the pH value of the prepared solution adjusted to 11 using NaOH solution (40 g/L) followed by 1 h agitation at ambient temperature. The obtained slurry was then passed through a sieve (80 mesh) to separate the solid part, followed by washing several times with distilled water and once more screening. The obtained supernatant was passed through a 200 mesh sieve to remove the fine solids. Finally, the supernatant sediment collected, washed several times with distilled water and then centrifuged at 4000× *g* for 10 min. The collected pellets were oven-dried at 60 °C, milled with Moulinex Grinder, passed through a 20 mesh sieve, and kept in sealed bags until use.

### 2.3. Determination of MIC and MBC of EOs

The minimum inhibitory concentration (MIC) and minimum bactericidal concentration (MBC) of the selected EOs against *Escherichia coli* PTCC 1330 (*E. coli*), *Staphylococcus aureus* PTCC 1764 (*S. aureus*), *Saccharomyces cerevisiae* PTCC 5269 (*S. cerevisiae*), and *Aspergillus flavus* PTCC 5006 (*A. flavus*) were determined by the microdilution broth method according to the procedure reported by Predoi et al. [23]. Briefly, 100 µL of each EO was dissolved in DMSO and then the 10 serial dilutions were prepared by microtiter plates containing MHB. Microbial suspensions of 2.0 × 10^8^ CFU/mL were obtained from the growth of solid cultures on TSA after 24 h. Then, the MIC and MBC were determined by the plating method. The lowest levels of the tested materials which visually inhibited the growth, and, respectively, determined 99.9% growth inhibition on MHA plates, were considered as MIC and MBC, respectively. Finally, the EO possessing the highest antimicrobial activity was loaded into the selected PVA-PBS blend film.

### 2.4. Film-Forming Procedure

The films were prepared using the casting method (wet method) as described by Jayakumar et al. [14] with some modifications. Neat PVA film was prepared by addition of 3 g PVA in 100 mL distilled water followed by heating at 80 °C for 6 h under stirring at 500 rpm. The obtained solution was then poured into a 15 cm diameter Teflon Petri dish and air-dried by oven at 60 °C for 12 h. The neat PBS film was also produced by the addition of 2 g PBS in 100 mL distilled water followed by heating at 95 °C for 1 h under stirring at 500 rpm. To prepare the blended films, different proportions of PVA to PBS were mixed together and dissolved in distilled water and heated for 60 min at 90 °C under stirring at 400 rpm. The treatments were named PV-PB (PVA-PBS) (50:50), PV-PB (60:40), PV-PB (70:30), PV-PB (80:20), PV-PB (90:10) and PV-PB (100:0), which show PVA films containing 50, 40, 30, 20, 10 and 0% PBS, respectively. In order to remove impurities and bubbles, the solutions were deaerated under a vacuum pump and then passed through Whatman filter paper No. 3. We added 10% glycerol to the filtrated solutions on a magnetic stirrer (400 rpm) for 30 min at 37 °C until obtaining a completely homogeneous solution. The samples were finally placed in an oven at 60 °C for 24 h and desiccated to obtain the desired blends for the films. Then, the best sample was selected from the above treatments and different essential oils were added to it. To fabricate antimicrobial biocomposite, 1, 2 and 3% (*v*/*v*) of CEO were loaded into the selected blended film solution. Tween 80 (10%) was used as an emulsifier to ease the CEO incorporation into the blended film. The film solution was homogenized at 15,000 rpm for 5 min using an UltraTurrax homogenizer. The prepared films were poured into 15 cm diameter Teflon dishes and left at 30 °C for 24 h in an oven. The dried films were peeled off from the surface and kept in a desiccator at 25 °C until evaluation.

### 2.5. Film Characterizations

#### 2.5.1. Thickness and Water Vapor Permeability

Film thickness was measured by using a digital micrometer (Mitutoyo, Tokyo, Japan) from different areas of the produced films. ASTM E96-00 was used as standard protocol to calculate water vapor permeability (WVP) [24]. Briefly, the obtained films were cut into 2 cm diameter discs and enfolded on top of the vial cells by parafilm, and finally moved to a desiccator saturated with K_2_SO_4_ to reach relative humidity (RH) of about 100% at ambient temperature. The WVP value of each film was calculated based on the weight gain of the permeation cells over time. To do that, the water vapor transmission rate (WVTR) was determined as the slope of the permeation (g/s) divided by the transfer zone (m^2^). By calculation of WVTR and film thickness, the WVP (g mm kPa^−1^ h^−1^ m^−2^) was calculated as follows:

(1) $$\mathrm{WVP}=\frac{\mathrm{WVTR}}{P\left(R_{1}-R_{2}\right)}\times X$$

where X is the film thickness, P is vapor pressure of water at saturation state (Pa), and R_1_ and R_2_ are RH inside of desiccator and permeation cell, respectively.

#### 2.5.2. Solubility

The solubility of films was determined according to Palma-Rodríguez et al. with slight modifications [25]. The film sheets were cut into 2 cm × 3 cm pieces and kept in a desiccator with RH of about zero over 7 days. The pieces were then weighted and transferred to water beakers containing 80 mL distilled water under agitation for 60 min at ambient temperature. The samples were placed in an oven at 60 °C until reaching a constant weight and the percentage of solubility was calculated as follows:

(2) $$\mathrm{Solubility}\;\left(\%\right)=\frac{\mathrm{Initial}\;\mathrm{dry}\;\mathrm{weight}-\mathrm{Final}\;\mathrm{dry}\;\mathrm{weight}}{\mathrm{Initial}\;\mathrm{dry}\;\mathrm{weight}}\times 100$$

#### 2.5.3. Water Absorption Capacity

Water absorption capacity (WAC) of films was determined according to the approach depicted by Lum et al. [26]. The initial weight of films (W1) was measured and the film samples were dipped in 30 mL of distilled water for 60 min. Afterward, the samples were removed from the water, the water remaining on the surface of films was absorbed by filter paper, and the wet films left at room temperature for 10 min before throwing away surface moisture and being weighed again (W2). The WAC of films was calculated according to the following equation:

(3) $$\mathrm{WAC}\;\left(\%\right)=\frac{W2-W1}{W1}\times 100$$

#### 2.5.4. Opacity

The opacity of films was measured according to Abral et al. [27] by using a spectrophotometric assay. Briefly, rectangular pieces of films (1 cm × 3 cm) were positioned inside the spectrophotometer (Shimadzu, Tokyo, Japan) cell and then the absorbance spectra in the range of 400 and 800 nm were monitored. The opacity value of films was measured based on the area under the absorbance spectrum.

#### 2.5.5. Mechanical Parameters

The tensile strength (TS), as maximum breaking force, and elongation at break (EAB), as deformation criteria, of films were measured as main mechanical parameters for packaging films according to standard protocols [28]. Films were cut into 1 cm × 10 cm strips and kept in a desiccator enclosing with NaBr solution with RH of 57% for 3 days before the test. A texture analyzer (SMT5, Santam, Tehran, Iran) equipped with 100 N load cell, 10 cm distance between grips, and the crosshead speed of 10 mm/min was used to determine the mechanical attributes.

#### 2.5.6. Microstructure Test

The surface morphology of the biocomposite films was observed by scanning electron microscopy (SEM) (Hitachi, Japan) at the accelerating voltage of 30 kV. The films were cryofractured by liquid nitrogen, coated with a gold layer using an ion-sputter coater, and finally visualized.

#### 2.5.7. X-ray Diffraction (XRD) Test

XRD test was performed by using a CuKα radiation source on a Philips PANalytical X’Pert diffractometer (Amsterdam, the Netherlands) at a voltage of 40 kV and 100 mA. The scans were plotted in a 2θ diffraction angle oscillating from 5–60° at the scan speed of 2 °/min with a wavelength of 0.154 nm.

#### 2.5.8. Fourier Transform Infrared (FTIR) Test

The FTIR test of biocomposite films was performed using a vacuum infrared spectrometer device (Billerica, MA, USA) through a KBr module. The spectra of the tested films were acquired at the wavenumber range of 4000–400 cm^−1^ and the resolution of 4 cm^−1^ with 16 scans at ambient temperature.

#### 2.5.9. Antimicrobial Test

The agar diffusion approach was used to determine the antibacterial effects of the produced films on *E. coli*, *Listeria monocytogenes* PTCC 1298 (*L. monocytogenes*), *Lactobacillus sakei* PTCC 1272 (*L. sakei*), and *Pseudomonas fluorescens* PTCC 1181 (*P. fluorescens*) bacteria. Briefly, the films were cut into 15 mm diameter discs and placed on BHI agar plates. The plates were inoculated with 0.1 mL of the broth culture of the mentioned bacterial strains overnight. The plates were then incubated at 37 °C for 24 h and the diameter of the inhibition zone was measured using a digital micrometer. The inhibition zone area was calculated after subtraction from the film disc area [2].

### 2.6. Statistical Analysis

Statistical analyses of the collected data were performed by SPSS V. 18.1. One-way analysis of variance (ANOVA) by Duncan multiple range tests were employed to figure out the significant differences between variables at the probability level of 5%. All tests were performed for least at three replications.

## 3. Results and Discussion

### 3.1. Characterization of the Blended Films

In the present study, primarily a number of key physical and mechanical tests were performed on biocomposite films obtained from PVA-PBS blend in order to select the best treatment. As shown in Table 1, the thickness of packing films was decreased by the addition of PBS, so that the thickness value decreased from 69.5 µm neat PVA film to 61.1 µm in PV-PB (50:50) film. The difference in thickness of the two biopolymers is due to the amount of dry matter used to fabricate the films, which results in a denser layer of film in the PVA film compared to the PBS film (3% *w*/*v* vs. 2% *w*/*v*, respectively). Accordingly, the presence of higher amounts of PVA in the film compared to PBS causes a thicker film layer. It has been reported that the thickness of natural films prepared for food packaging is in the range of 50 to 200 μm [6]. Therefore, the results obtained from the produced films are all in the appropriate range. The films obtained from these two polymers have a thickness of about 40 to 100 μm.

According to recent findings, carbohydrate-based biopolymers are generally not suitable barriers to the penetration of water vapor and other gases, while using other synthetic polymers, the addition of nanomaterials, crosslinking agents, and the use of other fillers can create a suitable barrier against steam or gas permeability [2,13]. According to Table 1, the neat PVA film showed a better potential for barrier formation against water vapor transfer compared to the blended films containing PBS so that WVP increased from 0.25 g mm kPa^−1^ h^−1^ m^−2^ to 0.42 g mm kPa^−1^ h^−1^ m^−2^ as a result of the incorporation of 50% PBS in PV-PB (50:50) treatment, while the addition of 10 and 20% PBS did not significantly affect the WVP value compare to the control film. In general, the higher the amount of PBS in the blended film, the lower the ability to prevent water vapor from penetrating the film. The differences in WVP of the prepared films are presumably related to the various hydrophilic natures of PBS and PVA polymers, both possessing high water affinity due to the presence of hydroxyl groups with different density and dissemination in their polymers chains which affect the mass transfer of water vapor molecules and water absorption capacity [29]. Similar results were reported by Gómez-Aldapa et al. [30] who correlated the superior barrier properties of PVA vs. potato starch to the well-ordered structure and presence of more hydroxyl groups in PVA film, improving the crystallinity and polarity of PVA-starch blend films.

Table 1 also shows the solubility of PVA film and PVA-PBS blend films. As observed, the addition of 50% PBS significantly decreased (*p* < 0.05) the solubility of PVA film from 66.2% to 42.8%. The addition of 10% PBS (PV-PB (90:10)) did not significantly change the solubility of the neat PVA film, while higher incorporation (20–50) declined the PVA film’s solubility. This can be associated with the formation of molecular interactions between PVA and PBS chains, limiting the water affinity [29,31]. This molecular interaction is mainly attributed to the generation of hydrogen bonds between amylose and amylopectin groups of starch and -OH groups of PVA, reoriented the hydrophobic groups of PVA polymer chain and consequently increased the hydrophobicity of the PVA-starch blend films [29] and decreased the amount of free -OH groups [32].

The results of the WAC values of the PVA film and PVA-PBS blend films are presented in Table 1. The incorporation of PBS within the PVA film significantly decreased the WAC of PVA film so that the incorporation of 50% PBS (PV-PB (50:50)) decreased the WAC of neat PVA film from 332% to 221%. PVA, per se, has a lot of hydroxyl groups as a water-soluble polymer and tends to absorb more water molecules in comparison with starch as a water-sensitive biopolymer [26]. These findings are in line with the results already obtained for the solubility of PVA-PBS blend films.

The opacity values of the films are also shown in Table 1. As can be seen, the addition of 10–20% PBS to PVA film did not change the transparency of the blended films compared to the control PVA film, while the incorporation of higher amounts of PBS significantly decreased the transparency of the blended films. The lower opacity of the neat PVA samples is probably due to the more uniform structure of PVA polymer itself, while the PBS biopolymers contain a lot of low-organized chains [29,31].

The mechanical properties of the packaging films are also considered as one of the main parameters affecting their use for industrial applications. According to the results, the film made of neat PVA has a TS of 52.6 MPa while addition of 10–50% PBS caused a significant decline in the TS of films so that the addition of 50% PBS decreased this value to 28.0 MPa (Table 1). The incorporation of more PBS content into the PVA films produced brittle structures without any considerable plastic deformation. These results are consistent with the results reported by Fortunati et al. [33]. Such high TS values in neat PVA films compare to those containing PBS, which is probably owing to the formation of stronger interactions between the PVA chains, while PBS films usually generate stiffer films with less stretchability.

On the other hand, PVA usually has the ability to produce ultraelastic (flexible) packaging films with high EAB values. The incorporation of 10% and 20% PBS into the neat PVA film did not alter the EAB value of the control films, while higher loading of PBS caused a remarkable decrease in the flexibility of the blended films. This is probably related to the presence of lower inter-molecular spaces between starch chains, which limits the free movement of molecular chains. The improvement of the mechanical attributes of the starch films by the addition of high molecular weight PVA is mainly related to the formation of more compressed crosslinked structures during the film fabrication process [30].

According to the results presented in Table 1, the mixture of PVA-PBS at the ratio of 80:20 seems to have suitable physical, barrier, transparency, and mechanical characteristics to fabricate packaging films for food applications. Therefore, considering the physical, mechanical, environmental, and economic aspects (due to the cost-effectiveness of starch films compared to synthetic polymers) PV-PB (80:20) was selected as the selected treatment for loading various essential oils.

### 3.2. MIC and MBC of EOs

The MIC and MBC of the EOs, as antimicrobial indicators, were determined and the obtained results are presented in Table 2. As observed, all tested EOs showed significant antimicrobial properties against a range of Gram-positive and Gram-negative bacteria in addition to fungi. CEO exhibited considerable antibacterial effects, especially against Gram-positive bacteria (*S. aureus*), so the MIC and MBC values were 1.56 and 3.12 µg/mL, while these values were 3.12 and 5.56 µg/mL for *E. coli*, respectively. In addition, the antimicrobial effectiveness of CEO against *S. cereviseae* was slightly higher than *A. flavus*. The antimicrobial activity of GiEO was slightly higher than GaEO. Accordingly, CEO was selected as the most efficient EO to boost the antimicrobial attributes of the selected film blend (PV-PB (80:20)).

### 3.3. Characterization of the Blended Films Containing CEO

#### 3.3.1. Physical Attributes

According to the results obtained by MIC and MBC tests, 1, 2 and 3% (*v*/*v*) CEO were loaded into the PV-PB (80:20) solution to fabricate CEO-loaded composites. Table 3 displays various physical properties of the blend biocomposite films containing various percentages of CEO. As can be seen, the film thickness significantly increased from 66.4 to 71.3 µm by increasing the CEO concentration up to 3%. The formation of intermolecular connections amongst PVA-PBS molecules was attenuated owing to the suitable compatibility of PVA-PBS and CEO over the hydrogen bonding, resulting in a loosed biocomposite structure and amplified biocomposite film thickness. Similar results of increasing the film thickness of pullulan-based biocomposites by incorporating 8% CEO are reported by Chu et al. [34].

From Table 3, it can be seen that the WVP value of the control blend film experienced a decreasing pattern when various concentrations of CEO were loaded within its matrix. Loading 3% CEO could decrease the WVP of control film from 0.29 g mm kPa^−1^ h^−1^ m^−2^ to 0.20 g mm kPa^−1^ h^−1^ m^−2^, leading to improved barrier properties. In agreement with the obtained results, several studies reported that the incorporation of CEO and other EOs results in obtaining biopolymers with improved permeability attributes [34,35,36]. The drop in the WVP value of biopolymers as a result of EO addition is mainly attributed to the hydrophobic nature of EOs, so that EO addition caused an increased traveling distance for water molecules to diffuse through the biopolymer matrix (Chu et al., 2019). Vahedikia et al. [2] also stated that incorporation of CEO into the zein film can create various covalent and hydrogen bonds among zein and CEO molecules, limiting the free hydrogen bonds and leading to a decreased WVP.

The water solubility of packaging films plays an important role in protecting food products during transportation and storage. The water solubility of the fabricated PVA-PBS-CEO biocomposites is presented in Table 3. A significant decrease in the solubility index of the control film was observed when various concentrations of CEO were loaded within the film matrix so that about an 18% decrease in solubility was recorded for the control film containing 3% CEO. A similar pattern was also reported for CEO-loaded chitosan, whey protein concentrate, and zein biocomposite films by Ojagh et al. [37], Bahram et al. [38], and Vahedikia et al. [2], respectively. An increase in film hydrophobicity as a result of the formation of crosslinked ester and/or amide groups due to the presence of CEO leads to an attenuated water affinity of the constructed biocomposites.

The WAC values of the PVA-PBS blend films containing CEO are displayed in Table 3. About 62, 102 and 147% reduction in WAC of the control film was occurred by adding 1, 2 and 3% CEO, respectively. The interaction between phenolic compounds of CEO and functional groups of PVA and PBS presumably decreased the availability of hydroxyl groups and hydrogen bonds, limiting the attachment of water molecules to the control film [39]. In agreement with the obtained results, incorporation of CEO and peppermint EO into the chitosan films showed an attenuated WAC [37,39].

The opacity (an index of the biocomposite appearance) values of the blended films containing CEO are also presented in Table 3. The addition of various concentrations of CEO into the control film could significantly increase the opacity value. This is probably due to the natural color of the CEO (yellow) and Tween 80 (as surfactant), and the formation of small CEO droplets throughout the film structure. The higher concentrations of CEO are capable of the formation of larger light scattering properties, generating a less transparent appearance [34].

#### 3.3.2. Mechanical Attributes

**Figure 1:**
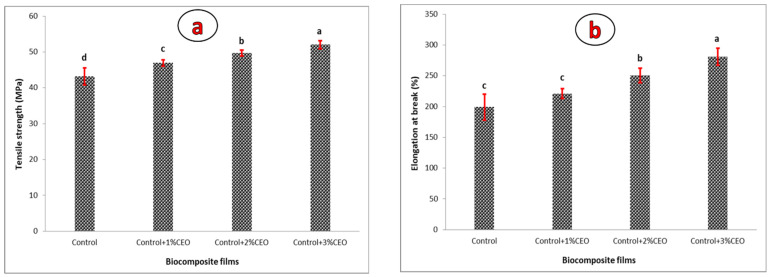
TS (**a**) and EAB (**b**) of PBS-PVA films containing 0–3% CEO.

Figure 1 demonstrates the EAB and TS values of the blended films containing CEO. Based on Figure 1a, the addition of 1–3% CEO to the matrix of PVA-PBS films could significantly (*p* < 0.05) improve the TS value, exhibiting the plasticizing effects of the added CEO-Tween 80. As observed, the TS values of the control film increased from 43.2 MPa to 46.9, 49.7 and 52.0 MPa in control films containing 1, 2 and 3% CEO, respectively. It has been stated that the formation of robust interactions between CEO and biopolymer via crosslinking effects of CEO components is capable of decreasing the free volume within the biopolymer structure, resulting in a denser network [37]. Another reason could be due to the lower moisture content of CEO-loaded films over the film-forming process which leads to the strain drop and then increase in TS value. Similar findings were reported by Vahedikia et al. [2] on CEO-loaded zein film, and Atares et al. [40] on CEO-loaded soy protein isolate film. The results of EAB changes are also plotted in Figure 1b. The CEO-loaded films showed significantly higher EAB values (more flexible films) compared to the control film so that the EAB increased from 197% in the neat control film to 281% in the control film containing 3% CEO. The CEO and Tween 80 within the film formulation can simply be deformed and enhance the film extensibility. Similar results were reported by Go and Song [41] who worked on hybrid chitosan films containing java citronella EO, and also by Han et al. [42] who worked on CEO-loaded sodium alginate-carboxy methyl cellulose films.

#### 3.3.3. Microstructure Analysis

**Figure 2:**
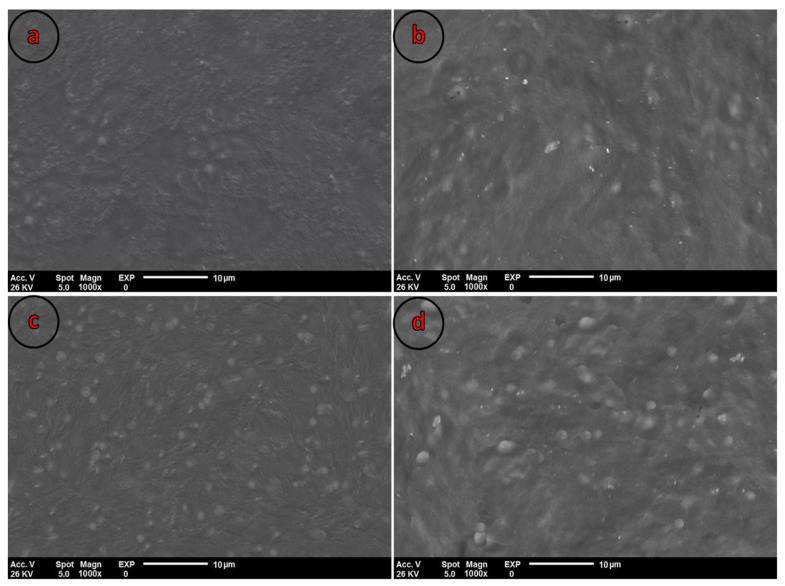
SEM micrographs of the control film (**a**), control film containing 1% CEO (**b**), control film containing 2% CEO (**c**), and control film containing 3% CEO (**d**).

Figure 2 displays the SEM micrographs of the control blend film and CEO-loaded biocomposites. A homogeneous interface without any pores was observed in the control film, revealing the appropriate blending of PBS and PVA chains in producing a film with high integrity. As can be seen, the microstructure of the control film was influenced by the incorporation of various concentrations of CEO so that some EO microdroplets were observed in CEO-loaded films. However, the CEO microdroplets were well-dispersed within the polymer matrix, most likely due to the action of Tween 80 as surfactant. In addition, no obvious coalescence and flocculation of oil droplets was found in the composite, demonstrating the suitable compatibility of the prepared formulation and the formation of hydrogen bonds between the CEO and the PBS-PVA blend. Similar findings were reported by Chu et al. [34] on the addition of CEO to pullulan films up to 8%, while higher concentrations caused significant structural disruptions within the prepared film. Such uniform distribution of CEO microdroplets could justify the modified permeability and mechanical attributes of the control film. Surfactant micelles are capable of encapsulating CEO microdroplets within the film matrix, boosting their antimicrobial and antioxidant capacities, and consequently providing a composite film with enhanced biological and structural attributes [43].

#### 3.3.4. XRD Analysis

**Figure 3:**
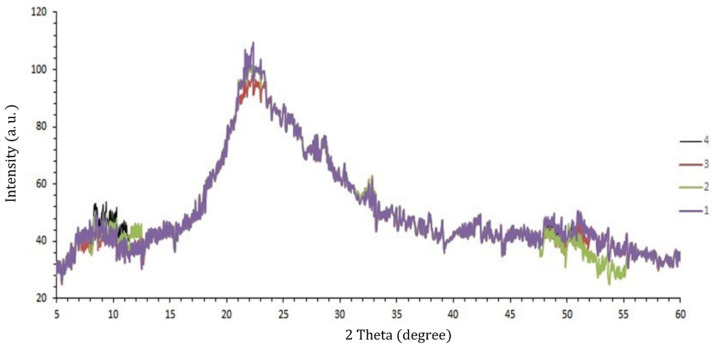
XRD patterns of the control film (1), control film containing 1% CEO (2), control film containing 2% CEO (3) and control film containing 3% CEO (4).

The X-ray patterns that show the crystalline and amorphous areas of the polymers structures are presented in Figure 3. The PBS-PVA control film showed two distinguished peaks at 2Ɵ = 4.395° and 2Ɵ = 22.309°, most likely due to the low crystallinity of the produced blended film. Two small peaks were also found at 2Ɵ = 18.4° and 2Ɵ = 26.9°. Incorporation of 1% CEO did not change the position of the peaks so that the similar peaks (at 2Ɵ = 4.973° and 2Ɵ = 22.228°) were found in the XRD pattern and small peaks were also remained unchanged. However, in 2% CEO-loaded films, the peak at 2Ɵ = 4.395° shifted right to 2Ɵ = 7.259° and the peak area enhanced significantly. Similarly, this peak shifted right to 2Ɵ = 7.102° in the biocomposite containing 3% CEO. These shifts signifying the disruption of the control film structure as a result of CEO addition. Other mentioned peaks did not shift in the control film or in the CEO-loaded films. Vahedikia et al. [2] reported that the XRD pattern of zein film did not change as a result of the addition of 2% CEO, while considerable changes were reported by Xu et al. [44] in chitosan-gum Arabic edible films containing 8% CEO.

#### 3.3.5. FTIR Analysis

**Figure 4:**
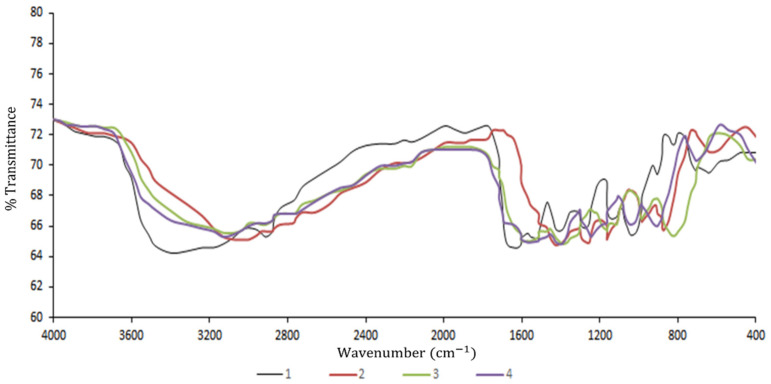
FTIR spectra of the control film (1), control film containing 1% CEO (2), control film containing 2% CEO (3), and control film containing 3% CEO (4).

Figure 4 shows the FTIR spectra of PBS-PVA blend film containing 0–3% CEO to investigate the intermolecular interactions. Significant differences were identified in the spectra profiles of the control film and those containing 1–3% CEO, while the spectra patterns on control films did not alter particularly in treatment loaded with 2 and 3% CEO. As observed, the O-H stretching bonds at 3431 cm^−1^, which are assigned to inter- and intra-molecular hydrogen bonds, experienced remarkable changes as a result of CEO addition, specifying the interaction of CEO with the matrix of PBS-PVA blend films through the hydrogen links. The spectra of the PBS-PVA film displayed some bands at 2911, 1618, 1423 and 814 cm^−1^, which could be assigned to C-H stretching, asymmetric COO^-^ stretching, CH_2_ bending, and C-O stretching vibrations, respectively [42]. As shown in Figure 4, considerable changes are observed in the intensity and peak area of the CEO-loaded films in comparison with the control film. Furthermore, a distinguished absorption band was observed at ca. 1392–1375 cm^−1^ in the CEO-loaded films, which could be associated with the distinguishing peak of CEO. Chu et al. [34] found such a distinguishing peak at 2375–2323 cm^−1^ and ascribed it to the presence of CEO within the pullulan structure.

#### 3.3.6. Antibacterial Analysis

Table 4 represents the antibacterial activity of CEO-loaded PBS-PVA blend films against various G-positive and G-negative bacteria. As observed, the CEO-loaded films showed significant antimicrobial effects (*p* < 0.05) on both G-positive and G-negative bacteria in the following order: *L. monocytogenes* > *L. sakei* > *E. coli* > *P. fluorescens*. The inhibition zone of the studied bacteria was increased by an increase in the CEO concentration within the control film. CEO-loaded films were more effective in controlling G-positive bacteria compared to G-negatives. The presence of several bioactive components, such as cinnamaldehyde, linalool, eugenol and 1,8-cineole, is attributed to the inhibition of various bacterial strains, most likely due to the prevention of protease and amylase enzymes by bacteria [2,37]. Higher antimicrobial attributes against G-positive bacteria could be associated with the intrinsic structural differences in the cell wall constructions of G-positive and G-negative bacteria. The cell wall of G-positive bacteria is mainly formed by a thicker layer of peptidoglycan and a small amount of proteins, while G-negatives possess thinner but more complex cell walls composed of peptidoglycans, lipids, polysaccharides, and proteins. It is worth mentioning that the G-negatives have an outer membrane layer covering the cell wall compartments [2].

## 4. Conclusions

In the current study, the blending of PVA and PBS composites was successfully performed and a part of PVA was substituted with a PBS biopolymer to achieve a semi-bio-based biodegradable composite film with improved structural and physicomechanical attributes. Furthermore, the incorporation of 1–3% CEO within the matrix of the selected film (PVA:PBS (80:20)) could modify some mechanical, physical, and antimicrobial properties of the fabricated films. Such functional active packaging films are suitable candidates for various food packaging and pharmaceutical applications due to their antibacterial activity against pathogenic foodborne bacteria. The films are ecofriendly materials and their compatibility with body organs could boost their applications for further biomedical uses, such as bone fixation screws, stent coating, and suture threads. It is worth mentioning that the production of functional biopolymer-based composites on an industrial scale needs more investigation to introduce them as promising alternatives for petroleum-based polymers.

## Figures and Tables

**Table 1:** Various physical and mechanical attributes of PVA-PBS composite films.

Treatments	Thickness (µm)	WVP * (g mm kPa^−1^ h^−1^ m^−2^)	Solubility (%)	WAC * (%)	Opacity (AUnm)	EAB * (%)	TS * (MPa)
PV-PB (50:50)	61.1 ± 1.1 ^c^	0.42 ± 0.03 ^a^	42.8 ± 1.1 ^d^	221 ± 16 ^d^	28.0 ± 0.4 ^a^	108.1 ± 10.9 ^c^	27.5 ± 0.9 ^e^
PV-PB (60:40)	62.4 ± 0.9 ^c^	0.37 ± 0.02 ^a^	46.9 ± 2.6 ^c^	248 ± 12 ^d^	26.9 ± 0.3 ^b^	136.2 ± 12.0 ^b^	32.8 ± 1.6 ^d^
PV-PB (70:30)	64.9 ± 1.0 ^b^	0.33 ± 0.01 ^b^	51.2 ± 2.7 ^c^	274 ± 9 ^c^	25.8 ± 0.4 ^c^	159.8 ± 17.6^b^	38.0 ± 1.3 ^c^
PV-PB (80:20)	66.4 ± 0.8 ^b^	0.29 ± 0.02 ^c^	58.0 ± 3.1 ^b^	297 ± 12 ^b^	24.7 ± 0.4 ^d^	198.9 ± 20.9 ^a^	43.2 ± 2.4 ^b^
PV-PB (90:10)	67.0 ± 1.3 ^ab^	0.27 ± 0.01 ^c^	64.1 ± 2.2 ^a^	310 ± 14 ^ab^	24.3 ± 0.6 ^d^	224.0 ± 19.7 ^a^	48.1 ± 3.2 ^ab^
PV-PB (100:0)	69.5 ± 1.2 ^a^	0.25 ± 0.02 ^c^	66.2 ± 3.0 ^a^	332 ± 15 ^a^	23.8 ± 0.5 ^d^	241.8 ± 22.9 ^a^	52.6 ± 4.1 ^a^

* WVP: water vapor permeability; WAC: Water absorption capacity; EAB: elongation at break; and TS: tensile strength. Values are presented as means ± std. deviation and values with the same superscript letters in each column are not significantly different (*p* > 0.05).

**Table 2:** The antimicrobial activity of various EOs on selected foodborne microorganisms.

Essential Oils	Antimicrobial Factors	*S. aureus*	*E. coli*	*S. cereviseae*	*A. flavus*
CEO *	MIC * (µg/mL)	1.56 ± 0.01 ^c^	3.12 ± 0.08 ^c^	5.20 ± 0.05 ^c^	6.25 ± 0.15 ^b^
	MBC * (µg/mL)	3.12 ± 0.03 ^B^	5.56 ± 0.05 ^C^	6.25 ± 0.20 ^B^	6.25 ± 0.10 ^B^
GiEO *	MIC (µg/mL)	6.25 ± 0.11 ^b^	8.33 ± 0.15 ^c^	8.33 ± 0.32 ^b^	12.50 ± 0.60 ^a^
	MBC (µg/mL)	12.50 ± 0.32 ^A^	10.28 ± 0.45 ^B^	12.50 ± 0.65 ^A^	12.50 ± 0.44 ^A^
GaEO *	MIC (µg/mL)	8.33 ± 0.24 ^a^	12.50 ± 0.40 ^c^	12.50 ± 0.38 ^a^	12.50 ± 0.71 ^a^
	MBC (µg/mL)	12.50 ± 0.39 ^A^	12.50 ± 0.51 ^A^	12.50 ± 0.27 ^A^	12.50 ± 0.55 ^A^

* CEO: cinnamon essential oil; GiEO: ginger essential oil; GaEO: garlic essential oil; MIC: minimum inhibitory concentration; and MBC: minimum bactericidal concentration. Values are presented as mean ± std. deviation and values with the same superscript letters (A, B and C for MBC, and a, b and c for MIC) in each column are not significantly different (*p* > 0.05).

**Table 3:** Various physical properties of the selected PVA-PBS composite film containing different concentrations of CEO.

Treatments	Thickness (µm)	WVP * (g mm kPa^−1^ h^−1^ m^−2^)	Solubility (%)	WAC * (%)	Opacity (AUnm)
Control	66.4 ± 0.8 ^c^	0.29 ± 0.02 ^a^	58.0 ± 3.1 ^a^	297 ± 12 ^a^	24.7 ± 0.4 ^d^
Control + 1% CEO	68.3 ± 0.5 ^b^	0.26 ± 0.01 ^b^	51.2 ± 1.6 ^b^	235 ± 17 ^b^	29.9 ± 0.6 ^c^
Control + 2% CEO	69.2 ± 0.7 ^ab^	0.23 ± 0.01 ^c^	45.2 ± 2.0 ^c^	192 ± 10 ^c^	32.6 ± 0.5 ^b^
Control + 3% CEO	71.3 ± 1.1 ^a^	0.20 ± 0.01 ^d^	40.4 ± 1.5 ^d^	150 ± 12 ^d^	36.8 ± 0.9 ^a^

* WVP: water vapor permeability; WAC: Water absorption capacity. Values are presented as mean ± std. deviation and values with the same superscript letters in each column are not significantly different (*p* > 0.05).

**Table 4:** Antibacterial activity of CEO-loaded PBS-PVA blend films against various G-positive and G-negative bacteria.

Bacteria	Treatments	Inhibition Zone (mm^2^)
*L. sakei* (G-positive)	Control	0 ^d^
	Control + 1% CEO	26.2 ± 1.2 ^c^
	Control + 2% CEO	29.9 ± 1.0 ^b^
	Control + 3% CEO	35.4 ± 0.5 ^a^
*L. monocytogenes* (G-positive)	Control	0 ^d^
	Control + 1% CEO	30.5 ± 1.0 ^c^
	Control + 2% CEO	37.9 ± 1.4 ^b^
	Control + 3% CEO	43.1 ± 1.4 ^a^
*E. coli* (G-negative)	Control	0 ^c^
	Control + 1% CEO	18.1 ± 0.7 ^b^
	Control + 2% CEO	22.9 ± 1.3 ^a^
	Control + 3% CEO	25.4 ± 1.5 ^a^
*P. fluorescens* (G-negative)	Control	0 ^d^
	Control + 1% CEO	15.2 ± 0.7 ^c^
	Control + 2% CEO	17.8 ± 0.6 ^b^
	Control + 3% CEO	20.1 ± 0.9 ^a^

Values are presented as mean ± std. deviation and values with the same superscript letters are not significantly different (*p* > 0.05).

## Data Availability

The data presented in this study are available on request from the corresponding author.
